# A Tragedy of Womanhood

**Published:** 1913-08

**Authors:** G. Henri

**Affiliations:** Paris, Ill.


### A Tragedy of Womanhood.

BY DR. G. HENRI, PARIS, ILL.

I have been more and more convinced that the real responsibility for the growth and continuance of the social evil lies, not with the women who are all too frequently driven into the underworld, and kept there by Pharisaical self-righteousness, but with the respectable (?) man who are the patrons and supporters of the traffic.

This view has been emphasized by a tragedy of womanhood, the last act of which drama has been staged before me.

Some years ago, in making a lecture on social purity before an audience of ladies, I was severely criticized by one of the women in the round-table discussion, with which I always close such an address.

She said that she did not believe all the strictures which I had made on the extent of the wrong. She said that while she did not doubt my honesty in my opinions she was assured that the extent of my investigations had made the bad side of society to loom unduly large to me.

Recently, this woman with her husband got their property tied up in unproductive investments to such an extent that they found themselves stranded in a large city, with their household goods in storage, and their finances at so low an ebb that they were forced to live in a rooming house.

The husband was tied fast with an idea of making some speculative deal that would retrieve their position, and seemingly failed to consider their present necessities, and the woman took a position as a saleswoman in a great department store, where she was paid $6.00 per week and a commission. She is highly educated and one of the most intensely honest, earnest women it has ever been my fortune to know. She abhors the conventional falsity of much of modern society, is vivacious, and a brilliant conversationalist.

The establishment in which she found employment is considered a model of its kind, and is a beautiful structure, while the management is noted for its magnanimous regard for the well-being of its employes, near 2000 in number. There is a rest room, and a hospital, matrons and a doctor, and, in the restaurant, daintily prepared food is served at cost; nothing seemed lacking for the fulfillment of a broad humanitarianism.

Now, allow me to quote from the letter that tells of the serpent, lurking in this Eden of commercialism:

"Dear Doctor: I owe you an apology for the strictures which I, in my ignorance, once applied to your strictures on modern life, for you did not tell us half of the horrible truth.

"I am home today, and my troubles are such that I know of no one to whom I dare turn for sympathy and advice. Were I to tell my husband, I dread to think what would follow. I am sick, heart, soul and body, with an awful burden, for which I see no end.

"When first I went to my place of employment I felt that I was among ladies and gentlemen. My first shock came when, near the holidays, I was feeling hurt that I could not make the usual presents to my dear friends, and I spoke of it to one of the girls, who was telling me of her gifts. She said, 'Haven't you a friend?'

"I noted her emphasis on the last word, and told her of my husband. She frankly and brutally laughed at my unsophistication.

"The buyer on this floor had been in the habit of stopping at my table when trade was slack, and I liked him; he was so appreciative, and liked so many of my favorite authors. Then, one evening, I was detained with a customer as the clerks were leaving, and when I went to the locker room for my wraps, the lights went out. As I hurried down the corridor for the elevator, this man met me in the narrow passage and tried to engage me in conversation, but I brushed past him. A few evenings later, the same thing occurred, only this time he caught me by the arm, and made his terrible meaning plain, but I tore loose, and escaped. The next morning I went to the superintendent with a bitter complaint, and was transferred to another floor. Here the troubles were worse. There was a bargain sale on dress patterns two days after I had commenced in the new department, and I went to the manager and asked that a gown be laid aside until Saturday, for I was sadly in need of it. He looked intently at me; then took a ten-dollar note with the remark, 'Girlie; no need to wait until Saturday.' I wanted to kill him, but I had to hold my position, and I asked him if many of his people were in insane asylums, and went back to my table with my soul on fire. I have not got the dress.

"There is one old man with beautiful silvery hair, which he wears long, whom I had considered nice, as he was so different, never attempting the liberties with the girls which had become so frightfully frequent, since my eyes had learned to see the terrible things which I had not conceived to be possible. Then one day when we were blocked in a crowded aisle he slipped his arm about my waist and lifted me clear off the floor. I struck and kicked him and struggled free; then toward evening, where there was a lull in trade, he came to my table and, after an apology, he said, 'My God, I would give anything if you would allow me to demonstrate my love for you, and make life easier for you.'

"I rebuked him and defied him to have me discharged, but every day I see him and the wretch who offered me the money, watching me as a cat does a mouse.

"You may wonder why I do not quit, but what can I do when I have no place else to go, and the work is so necessary, merely to exist. When there is a slack week, and the commissions are short, there have been days at a time when a single cup of tea and a sandwich have been my breakfast, repeated for luncheon, and at these times some of the men, oh, so often, ask me to come to lunch with them; they appear to know the conditions.

"One week I had been ill, and lost some of the time, and I had come home weak, tired and hungry. A member of the firm had stopped at my table that afternoon, for the day had been stormy and dismal, and there was little trade. He had mentioned a play that was running at one of the principal theaters, and which happened to be a favorite of mine.

"We discussed the actors whom we had seen in this production, and he asked me if I were going to see it while in the city. I scornfully asked how he expected me, on my meager wages, to buy a ticket, when he asked to be allowed to escort me to the play and to go out to supper after. I declined with warmth, and he apologized, and then said that at any time, if I should reconsider the matter, I could call him at his hotel.

"When I reached home and paid my week's room rent, I had just 89 cents left, and, as I stood looking at the miserable coins, I felt desperate and damned. You have seen those who looked from a high tower on a cliff who felt a mad impulse to leap over, and so, for a moment, I reeled above the deadly abyss, for I started to the phone to call him, when with a shudder I realized and drew back.

"Then I went down onto the street, and to the old Jew, and the diamond papa gave me when I graduated went to join my wedding and engagement ring; pawned for the bare necessities of animal existence.

"Do not, my true friend, blame me too severely, that, for a moment, I stumbled, for it was physical weakness and weariness, hunger not alone for food, but as well for the intellectual life that means so much to me.

"Remember, that I was able to throw off the momentary weakness, and so passed safely what would have been immeasurably worse than death.

"There is a floor walker on this department who is a handsome fellow, whom the girls call 'the sweet singer,' and who has a fine voice, which he employs in evenings in a moving picture show. One day, in passing my table, he handed me a note, which read, 'My love for you is as a flaming sword, and your love for me would prove an armor against all the world.' I called him back as soon as I had read it, and demanded where he had copied such nonsense, and he told me that he has written it for 'Just You.' I asked what under God's heaven he meant, and he commenced something about my 'charming personality,' when I stopped him, and told him what a brute he was, but I was so upset that I had to go to the rest room and lie down for the remainder of the day.

"Now, as he passes the aisle, he hums 'Il Trovatore,' and looks at me as though he would like to carry me off, which he doubtless does.

"There is one elderly man who is a fellow countryman of mine whom I have known for the three months that I have been working here, and he has always treated me with the respect that I feel my due. Yesterday I was ill, and at 5 o'clock I asked to be allowed to go home. When I came to the exit, he was waiting, and, telling me how he had noted how ill I was, he wished to be allowed to escort me home. I gladly accepted the offer, for I counted him as the only manly man in a bunch of brutes. Arriving at my room, I invited him in, as I wished to have him meet my husband. No sooner was the door closed than he, too, declared that passion, which, with these creatures, seems to take the place of love. I ordered him to leave, but he begged forgiveness, and stayed until my husband came, when, having introduced them, I fled to the bathroom in tears.

"You can not conceive how I was humiliated and hurt, and yet I must bear it, for we must eat, and we must have a shelter.

"There are numberless petty insults, and there are the horrible moments when the cages of the elevators are packed until not one more can find entrance, when just 'working girls' and man brutes are crowded, and one's person is intentionally squeezed and handled as we go from the cloak rooms to the exit. I am almost exhausted, not so much with the work and the privations as by the conflict of a woman's soul, and heart, and will, to keep pure and clean against the hypnotic watchfulness of the human hyenas who smile upon me.

"Why, in God's name, will they not leave me alone, and seek those who are not unwilling, for, strange as it may seem to you, there are women who work on this floor with me who are hatefully jealous of these attentions to which I have been forced to submit.

"I have tried to find another line of employment, unsuccessfully, and I now know that other similar establishments are even worse than this.

"I can not go to my own relatives for help, for they refuse to come to my aid, unless my husband shall get back into business and do what they want. Tell me what I shall do."

Such, in part, is the letter.

Woman is considered legitimate prey far more frequently than the world at large knows, and her conquest is the king of sports for many who rank as of the highest respectability.

Were this woman in her proper sphere, the devils who are hounding her would not dare lift unholy eyes to her, and the fact that she is superior in virtue and in intelligence but adds zest to the game for her pursuers, just as the huntsman will suffer hardships to pursue the wily fox, the while he passes pussy basking on the garden wall without a thought.

The men who are engaged in this hellish sport—God save the term—usually hunt in packs, as do the hyenas that rifle graves.

It may be accepted as certain that there are numerous wagers among her tormenters as to which shall win the victim, and doubtless the plans are laid for a champagne supper for the victor, and that in this orgy it is expected that the guest of the evening, when flushed with wine, shall narrate the hideous incidents of the final surrender, whether the end came with tearful, shrinking pleadings, or whether the leash having slipped like the ziamph of Salbammbo, she yielded with the opposite extreme of desperate abandon, and the acclaim awarded the narrator will be measured by the grossness with which he paints the story in all its damning details.

The stories of the nameless debaucheries of men whom the world holds in highest regard, as shown in the Thaw trial, are of hourly occurrence. You ask what I shall do about this.

When I had read the pitiful letter, I called over the long distance phone and offered financial assistance, which she refused. In a voice indescribably weary, she said, "This is my fight; the battle of a woman's heart and soul, and I must win it alone; I only wanted the sympathy of someone who could understand, but sometimes, oh, dear God, I get so rebellious and desperate, and then I wonder, wonder, what will be the end."

And I, too, wonder what is to be the end; not for this particular woman, nor for any woman, but for the race and nation where the demand of men of fair fame, in the world's eye, keeps ever increasing its hellish demand for womanly purity as its foul plaything.

I have entitled this paper a "Tragedy of Womanhood," and the story of this woman, as I know it, preserves the dramatic verities, since the episode which I have related is the fourth act of a life drama.

The first was set as a pastoral, when, a score of years ago, she stood at the altar where there was all regard for the ecclesiastical side of the transaction, while the real purpose of the union was not even thought of—the procreation of children—for which this woman is most superbly equipped, since with all the depth of an intense, strong nature is coupled a wonderful love and appreciation of childhood. Indeed, my introduction to the inner life of this pair was when I was consulted as to the cause of her sterility.

This woman, with the strongest maternity and the deepest delight of whose existence is in flowers and beautiful things, will never have her rights, since the Episcopal marriage service was to her the portal of a living death.

The husband had sowed his wild oats, and the vas deferens had been closed by the inflammatory sterilization that had followed a youthful gonorrhea, though he was in ignorance of the fact that his marriage was but a farce, in so far as its real purpose was concerned.

The second act came a few years ago, when he returned home from an extended business trip, suffering with a violent attack of gonorrhea, which his local physician was unable to control, so that I was consulted by mail and the treatment of which brought out the story of the early disease, while the microscope during the treatment told of the absence of spermatozoa.

The wife accepted his explanation that he had been infected sleeping between dirty sheets, and condoned this direct insult to her womanhood. The crowning infamy of it all was found in what would class of the third act of the tragedy.

Shortly after she had commenced work, she had called me over the telephone. She told me that she had missed one of her menstrual periods and that, when she had told her husband, he had accused her of being unfaithful, citing the results of the microscopic findings, which he had not concealed from her and had heaped insults upon her until she had rushed from the rooms which sheltered them, and walked the street for hours, until she had thought to call me.

She wanted to know if it were possible that her husband had recovered from his disability, so that her hopes of other days were likely to be fulfilled.

I told her that this condition was impossible, and she said, "Thank God, when he made that awful charge, something snapped within me, and the thought of motherhood is now as hateful as it has been desired."

I referred her to a lady physician whom I knew to be tender, sympathetic and competent, and the trouble was soon explained as resulting from the unwonted exertion and some exposure, and the mind of the husband was set at rest.

Here was the worst example of the injustice of the double standard in sex accountability that I have ever met.

This woman's life has been made empty of children's prattle through his incontinence, then the later violation of his marriage vow, with its dire insult to her purity, and she had forgiven without question. But, when he, possibly measuring her by his own standard, imagined that she too had sinned, he at once exercised his masculine prerogative by abusing her. And the woman, pleading "duty," clings to him and the resultant burden.

When I thought to tell her that she had passed the line of duty, in the way that she is pursuing, she told me how he loves her; so much so that he is frantic when she is absent from him more than a day, and that now he frequently comes to her place of employment to get a few minutes of conversation.

It may be love that prompts him, but, if so, it is a love that I am glad that I can not comprehend.

And all the elements of this terrible tragedy are found on a perverted sexual standard, with the innocent one the worst sufferer, as ever happens in the wake of the evil of debased sexuality.

---

Make Drinking Odious.—Mr. Roosevelt deems the practice of drinking disgraceful, and, to be accused of it, a libel. In this contention, the courts sustained him. The President and the great Secretary of State share this view of it, and drink should be abolished everywhere, as it is at the White House. There is no kind of excuse for it, and, were it abolished, there would be fewer chronic diseases—fewer premature deaths—deaths of splendid men scarcely reached middle life,—you can all recall such men, cut off at fifty or less,—with Bright's disease. There would be less insanity, less crime, fewer divorces, less poverty, less prostitution, less human misery. Think of a fuddled engineer in charge of a train of precious lives. It is truly astonishing that sensible men will continue to poison themselves. The saloon must and surely will go;—it belongs to a past age.

